# Comparison of two novel methods for counting wheat ears in the field with terrestrial LiDAR

**DOI:** 10.1186/s13007-023-01093-z

**Published:** 2023-11-26

**Authors:** Yangyang Gu, Hongxu Ai, Tai Guo, Peng Liu, Yongqing Wang, Hengbiao Zheng, Tao Cheng, Yan Zhu, Weixing Cao, Xia Yao

**Affiliations:** National Engineering and Technology Center for Information Agriculture (NETCIA), Zhongshan Biological Breeding Laboratory (ZSBBL), MARA Key Laboratory for Crop System Analysis and Decision Making, MOE Engineering Research Center of Smart Agriculture, Jiangsu Key Laboratory for Information Agriculture, Nanjing Agricultural University, One Weigang, Nanjing, Jiangsu 210095 People’s Republic of China

**Keywords:** Ear number, LiDAR, Density-based spatial clustering based on the normal (DBSC), Voxel-based regional growth (VBRG)

## Abstract

**Background:**

The metrics for assessing the yield of crops in the field include the number of ears per unit area, the grain number per ear, and the thousand-grain weight. Typically, the ear number per unit area contributes the most to the yield. However, calculation of the ear number tends to rely on traditional manual counting, which is inefficient, labour intensive, inaccurate, and lacking in objectivity. In this study, two novel extraction algorithms for the estimation of the wheat ear number were developed based on the use of terrestrial laser scanning (TLS) in conjunction with the density-based spatial clustering (DBSC) algorithm based on the normal and the voxel-based regional growth (VBRG) algorithm. The DBSC involves two steps: (1) segmentation of the point clouds using differences in the normal vectors and (2) clustering of the segmented point clouds using a density clustering algorithm to calculate the ear number. The VBRG involves three steps: (1) voxelization of the point clouds, (2) construction of the topological relationships between the voxels as a connected region using the k-dimensional tree, and (3) detection of the wheat ears in the connected areas using a regional growth algorithm.

**Results:**

The results demonstrated that DBSC and VBRG were promising in estimating the number of ears for different cultivars, planting densities, N fertilization rates, and growth stages of wheat (RMSE = 76 ~ 114 ears/m^2^, rRMSE = 18.62 ~ 27.96%, *r* = 0.76 ~ 0.84). Comparing the performance of the two algorithms, the overall accuracy of the DBSC (RMSE = 76 ears/m^2^, rRMSE = 18.62%, *r* = 0.84) was better than that of the VBRG (RMSE = 114 ears/m^2^, rRMSE = 27.96%, *r* = 0.76). It was found that with the DBSC, the calculation in points as units permitted more detailed information to be retained, and this method was more suitable for estimation of the wheat ear number in the field.

**Conclusions:**

The algorithms adopted in this study provide new approaches for non-destructive measurement and efficient acquisition of the ear number in the assessment of the wheat yield phenotype.

## Introduction

Wheat is the largest cereal crop under cultivation and is one of the three major food crops in the world. Hence, it plays a very important role in agricultural production [1]. Accurate and rapid monitoring of the growth of wheat is of great importance for predicting crop yield. Three key metrics for assessing wheat yield are the ear number per unit area, the grain number per ear, and the thousand-grain weight [2]. In particular, the ear number per unit area contributes the most to yield [3]. Increasing the ear number is an effective means to tap the yield potential of wheat and increase the yield. Therefore, a real-time, accurate and nondestructive estimation of the wheat ear number in the field can provide a scientific basis for predicting the yield of wheat and selecting wheat cultivars with high and stable yields. At present, remote sensing based on RGB images is widely used for the monitoring of wheat ear numbers [4]. Zhou, et al. [5] combined high-resolution, large-scale RGB images and multispectral images of wheat in the field to achieve high-throughput nondestructive field measurement of the wheat ear number. Additionally, it has been found that there are differences in temperature between the ears and other organs in wheat. Fernandez-Gallego, et al. [6] used a handheld thermal infrared instrument to measure the temperature distribution image of wheat in the field at the late-filling stage, and was able to separate the ears from the environmental background, and count them using the contrast histogram equalization method. However, such passive remote sensing methods are vulnerable to illumination conditions, background reflection, and vegetation structure. Many researchers have used deep learning to improve these shortcomings, but the deep learning approach needs to be based on a large amount of data, and it is challenging to build appropriate datasets [7–9]. In addition, image-based methods are unable to obtain fine three-dimensional (3-D) structural information from vegetation and suffer from more serious occlusion problems.

Light detection and ranging (LiDAR), as an active sensing technology, could record the 3-D structural morphology of an object by generating a point cloud through laser pulses [10]. Short wavelength laser radiation can penetrate the vegetation canopy to characterize the internal structure of the vegetation, thus overcoming the shortcomings of optical imaging techniques [11]. However, compared with the well-developed image processing technology of optical imaging, the application of LiDAR for extraction of crop ear number features in the field is still in its infancy. Saeys, et al. [12] employed two types of LiDAR sensors on a harvester to scan manually constructed, mature wheat plots of different planting densities. The effect of various traversing speeds on the estimation of the wheat canopy density was explored by fitting the positions of the wheat ear using a thin plate smoothing spline. Velumani, et al. [13] used two different point cloud segmentation algorithms, voxel-based segmentation and mean shift segmentation, to isolate and cluster the point clouds of ears in the wheat canopy in the field to estimate the ear numbers. For sorghum, which is taller than wheat and has clear ear characteristics, Malambo, et al. [14] extracted features of sorghum ears at maturity by density clustering based on single station terrestrial LiDAR point cloud data and measured the length and width of individual ears. Due to the conical view and the limited mounting height of ground-based LiDAR, dense vegetation canopies can be better observed from the zenith direction close to the ground [10]. Therefore, Blanquart, et al. [15] explored the use of various scan angles in LiDAR for estimating the wheat ear number in the field. The results showed that both the instrument height and the scan angle affected the accuracy of the estimation; however, the height of the instrument had a greater effect on the accuracy.

In summary, the methods for estimating the wheat ear number based on point cloud data can be divided into two main approaches: point cloud extraction and point cloud clustering. The ear point cloud is generally extracted and clustered separately according to the difference in the point cloud densities between the ear and other organs [13, 14]. In addition to the differences in the densities of the point clouds for the ear and the other organs, there is also a clear morphological difference between the ear and the other organs. The ear grows on an upright stem, and the leaves are continuously curved surfaces; thus, the normal vector can be used as an indicator of the stem and leaf segmentation of the crop [16]. In previous studies, the density of the point cloud was mostly used to set the segmentation threshold [10, 12]. However, in those algorithms, point clouds were underutilized to obtain information about crop structure without consideration of morphological differences among stems, ears, and leaves.

In addition to the density features, the 3-D spatial coordinate information of the point cloud is also an important feature and includes the spatial correlation information. To describe the spatial correlation of the discrete point clouds while reducing the amount of data, the discrete point clouds are mostly integrated into connected regions (i.e., point cloud voxelization) for spatial operations in forestry applications [17]. Velumani, et al. [13] coded the voxels to characterize the spatial correlation between the voxels and segmented the wheat ear point clouds. However, their study only involved wheat data from three microplots (10 m × 2 m). Therefore, the generality and universality of the algorithm under different growing conditions need to be further verified.

To rapidly and accurately determine the wheat ear number in the field, the present study entailed developing a density-based spatial clustering technique based on the normal (DBSC) and voxel-based regional growth (VBRG) algorithms in conjunction with terrestrial laser scanning (TLS) point cloud data to take advantage of the morphological characteristics of wheat. The DBSC method is based on points for direct data processing and parameter extraction, while the VBRG method is based on connected regions consisting of voxels for area operations. In this study, the effect of the differences in wheat growth on the algorithm was studied by setting up different experimental conditions to test the generality and practicality of the algorithm. In addition, the advantages and disadvantages of the two algorithms were compared with the aim of finding a more suitable method for estimating the number of wheat ears in the field.

## Materials and methods

### Experimental design

**Fig. 1:**
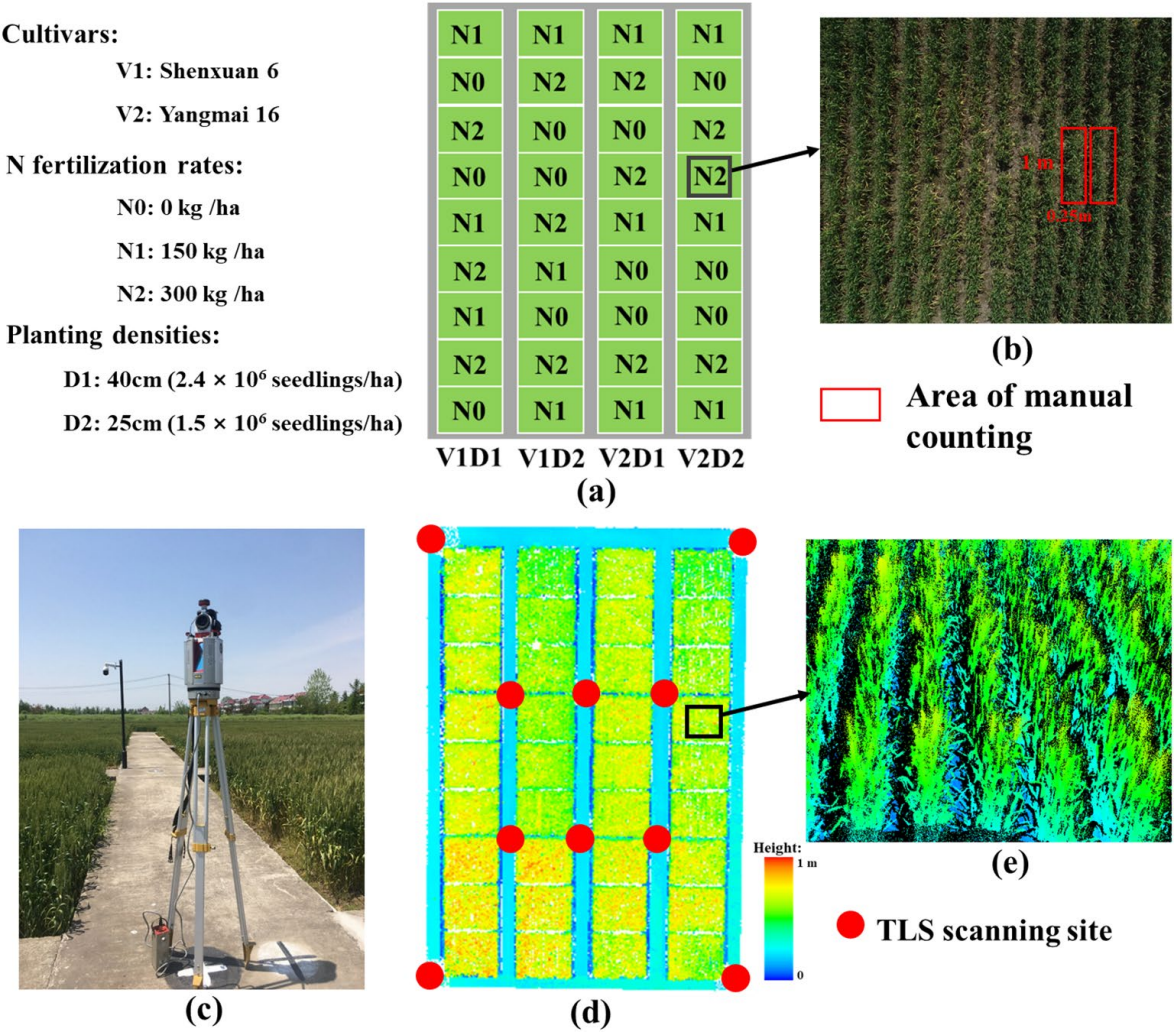
**a** The field experimental design of the wheat; **b** Image of wheat in the region of interest, with the red area indicating the area of manual counting of the ear number; **c** RIEGL-VZ 1000 device operating in the field at Rugao; **d** TLS data for the whole experimental area at the heading stage as displayed in the supporting software RiCSAN PRO; **e** Point cloud for wheat in the region of interest

The study was conducted at the experimental station of the National Engineering and Technology Center for Information Agriculture (NETCIA) located in Rugao, Jiangsu Province in eastern China (120°45′E, 32°16′N) during the winter wheat season of 2018 to 2019. Two wheat cultivars, ‘Shenxuan 6’ (V1) and ‘Yangmai 16’ (V2), were selected as being representative of compact and diffuse plant types, respectively. Three nitrogen (N) fertilization rates of 0 kg/ha (N0), 150 kg/ha (N1), and 300 kg/ha (N2) were established, among which N1 was consistent with the average nitrogen level. For each fertilizer regime, fifty percent of the N fertilizer was applied on the day of sowing, and 50% was applied at the jointing stage. Two planting density levels were set for the experiment: 25 cm (2.4 × 10^6^ seedlings/ha) for D1 and 40 cm (1.5 × 10^6^ seedlings/ha) for D2. For each set of growing conditions, the experimental design was established for split blocks with three replicates, for a total of 36 plots, each of which had an area of 30 m^2^ (6 m × 5 m) (Fig. 1).

### Manual measurements

It is difficult and time-consuming to count all the ears in each plot by manual counting. Therefore, we selected two rows with a length of 1 m and a width of 0.25 m as the manual counting area (Fig. 1b). The area chosen needed to be consistent in growth status with the whole plot. We determined the number of wheat ears in those two rows by manual counting and averaged each row. The wheat ear number for the whole plot was determined by accounting for the total area. Manual measurements were performed at four stages (heading, anthesis, early-filling, and late filling), and terrestrial LiDAR measurements were collected simultaneously.

### TLS measurements and data processing

The TLS instrument used in the study was a RIEGL-VZ 1000 system (RIEGL, Austria, https://www.riegl.com), which is a pulsed 3D scanner that emits near-infrared laser radiation. The specifications of the RIEGL-VZ 1000 system are listed in Table 1. To mitigate the effects of occlusion and to obtain a uniform point cloud density, a multiple-scan strategy was employed in all the trials. A 10-site scanning strategy was used with a scanning mode of 60° (an angular resolution of 0.06°) (Fig. 1).

**Table 1:** The specifications of the RIEGL-VZ 1000 system

Parameter	Value
Scanning principle	Pulse type
Laser wavelength	1550 nm
Maximum distance	1400 m
Pulse repetition rate	3 × 10^5^ pulses/second
Scanning accuracy	5 mm @ 100 m
Scanning range	360° × 100°
Beam divergence	0.12 mrad
Weight	9.8 kg

The preprocessing of the TLS data was conducted by the scanner bundled software RiSCAN Pro. The registration of the coordinates was the first step. An iterative closest point (ICP) algorithm was applied to register each independent scanner coordinate to the same reference coordinate system. The ICP algorithm calculated the transformation matrix by the least-squares method based on the corresponding points [18]. The coordinate registration was completed with an average error of 0.006 m for each campaign. Abnormal floating points caused by insects or airborne particles were removed manually. The wheat point clouds were then intercepted in the manually counted areas of each plot. Finally, the data for each area were exported into separate files.

### Methods for estimating the ear number

#### Density-based spatial clustering based on the normal

**Fig. 2:**
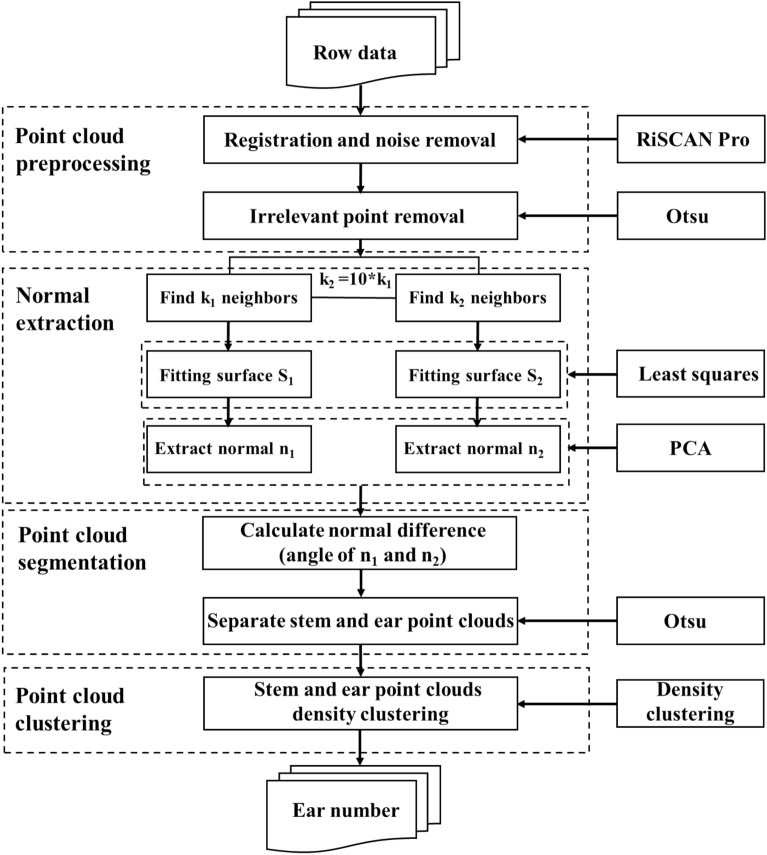
Workflow for estimating the ear number with DBSC

The algorithm of the density-based spatial clustering based on the normal (DBSC) includes three parts: point cloud preprocessing, ear point cloud segmentation, and ear point cloud clustering. First, the noise and irrelevant points in the point clouds were removed by the preprocessing step, then the leaf point clouds were divided from the stem & ear point clouds, and finally, the divided stem & ear point clouds were clustered as a function of density, and the ear number was counted (Fig. 2).

1. Point cloud preprocessing

**Fig. 3:**
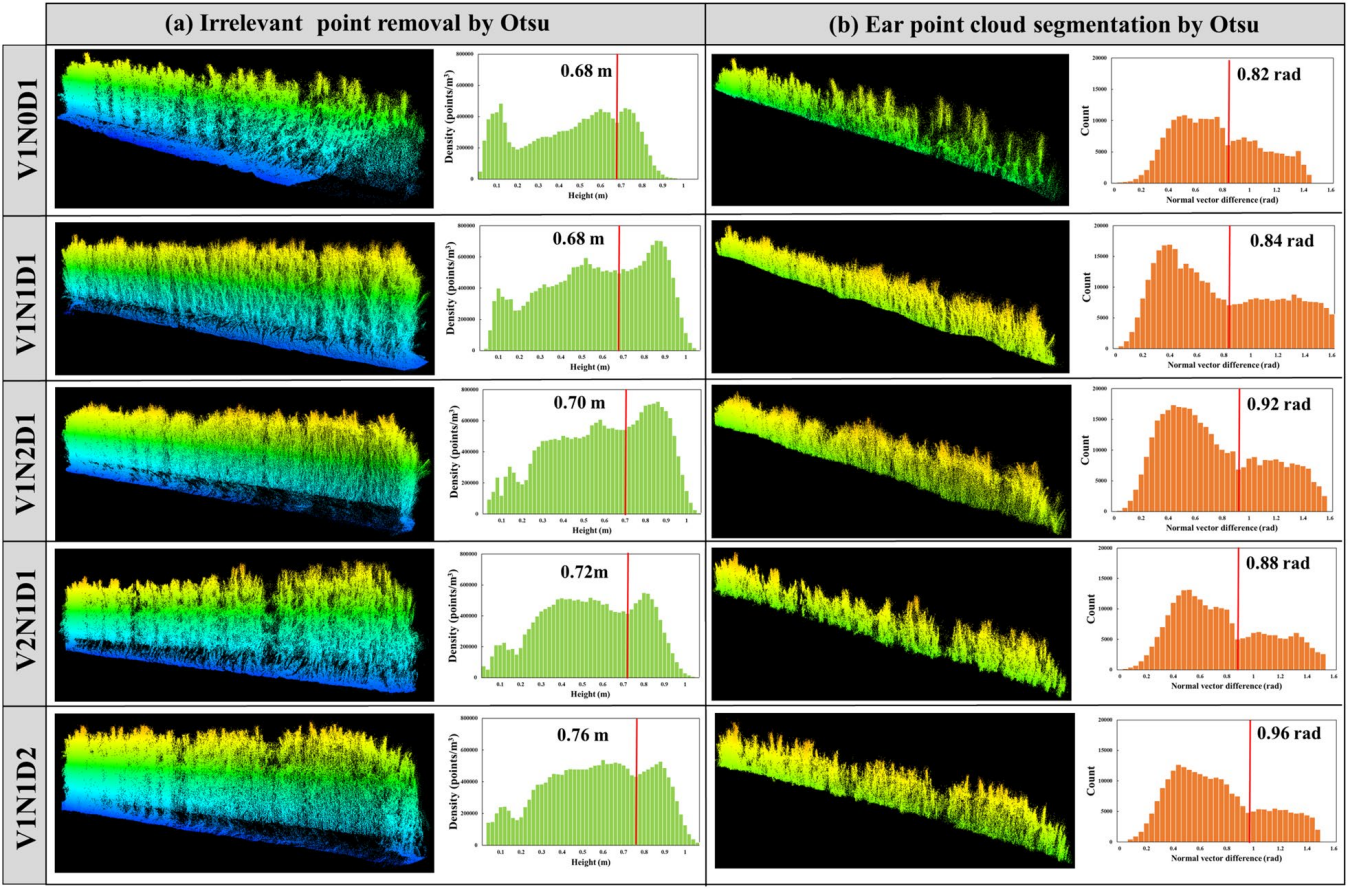
Schematic diagrams of removing irrelevant points (**a**) and segmenting ear point clouds (**b**) using the Otsu method. Subplots (**a**) and (**b**) show the histogram distribution of the point cloud density and the normal vector difference of the two cultivars (V1 and V2), N fertilization rates (N0, the N1, and N2) and planting densities (D1 and D2) for wheat during the early-filling stage

The DBSC preprocessing method includes not only the usual alignment and denoising of the point clouds but also the removal of irrelevant points. The irrelevant points are point clouds in the middle and lower parts of the plant, where the probability of wheat ears appearing is low. The removal of irrelevant points requires determining a cutting height “h” and removing the point clouds below “h”. The value of “h” is determined by the self-adaptation threshold method of Otsu [19]. In this algorithm, the points in each 2 cm height layer serve as the x-axis, and the height values are used as the y-axis to make a histogram of the point cloud density distribution (Fig. 3a). The occlusion causes a loss of point cloud information for the stem and leaf in the lower and middle sections of the wheat canopy. Therefore, the point cloud density in the middle and lower sections is smaller than that in the upper section where the ear is located, resulting in different density peaks for the point clouds. In the Otsu method, the value of the trough is located between the two peaks within the histogram and the corresponding height value is used as the cut height “h”.

(2) Ear point cloud segmentation

Wheat ears and stems and leaves exhibit distinct differences in morphology. The ear grows on an upright stem and remains upright during the late-filling stage, while the leaves are continuously curved. Thus, there is a large difference in curvature between the ear and leaf. The normal direction can be represented as a line on the surface that is perpendicular to the tangent plane of the surface at a point. The normal angle corresponds to an angle that is between the normal and the direction perpendicular to the horizontal. This angle reflects the magnitude of the curvature of the surface at a point. Based on an analysis of the wheat morphology, an edge-based segmentation algorithm was selected to segment the ear point cloud from the original point cloud using the difference in normal angles for the stem and leaf as the differentiation index.

**Fig. 4:**
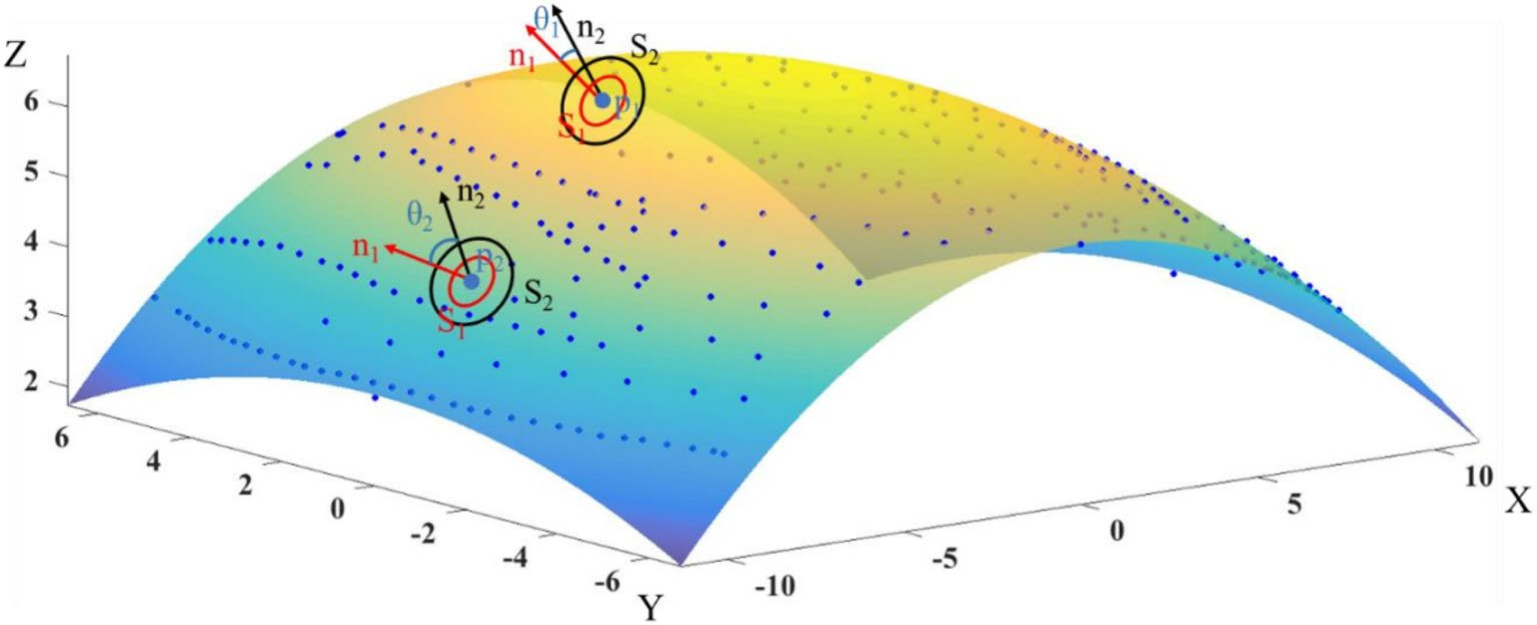
Schematic of the normal vector variance *θ* (*p*_1_, *p*_2_ are data points; the ellipses represent the fitted planes *S*_1_, *S*_2_; the arrows represent the corresponding normal vectors *n*_1_, *n*_2_ for the plane; *θ*_1_, *θ*_2_ are the corresponding differences in degrees between the normal vectors)

Individual points by themselves do not carry surface orientation or normal information. However, it is possible to estimate normal angles for discrete point clouds within a point cloud. For this, we select k points in the neighbourhood of the centre point (arbitrary point) to fit the surface, construct the local surface, and extract the surface normal. The following algorithm adopts the least-squares surface fitting method to construct the surface [20]:

1 $$S(n,d)=\mathrm{arg}min\sum_{i=1}^{k} {\left(n{p}_{i}-d\right)}^{2}$$

where *S* is the fitted surface, *n* is the surface normal vector, *p*_*i*_ represents point i, and *d* represents the distance of *p*_*i*_ from the origin of the coordinate (Fig. 4). The surface size changes as the number of points *k* changes. Two values of* k* with a tenfold difference in size (*k*_2_ = 10*k_1_) are typically chosen to obtain two fitted surfaces *S*_1_ and *S*_2_ with different sizes (*S*_1_ < *S*_2_). The smaller area surface S1 is contained within the larger area surface S2. The difference between the normals of S1 and S2 can be determined by calculating the differences among the normals of all points in the range of S1, which can then be applied in the next step of the cluster analysis.

In the present study, normal extraction was performed by principal component analysis (PCA), which analyses the eigenvectors and eigenvalues of a covariance matrix composed of study points and points in the nearest domain [16, 21, 22].

2 $$M\, = \,\frac{1}{k}\sum\limits_{i = 1}^{k} {\left( {p_{i} - \overline{p}} \right)} \,\left( {p_{i} - \overline{p}} \right)^{T}$$

3 $$\overline{p}_{i} \, = \,\frac{1}{k}\,\sum\limits_{i = 1}^{k} {p_{i,j} }$$

where $M$ is the covariance matrix, and $\overline{p}$ is the centre of gravity of the surface *S*. The normal vector *n* is the third eigenvector of the covariance matrix.

The angle *θ* between two normal vectors (*n*_1_, *n*_2_) with different directions is defined as the normal vector difference:

4 $$\theta = {\text{arccos}}\frac{{\left[ {n_{1} ,n_{2} } \right]}}{{\left\| {n_{1} } \right\|\left\| {n_{2} } \right\|}}\left( {0,\pi } \right)$$

where *n*_1_ and *n*_2_ correspond to the surface normal vectors of surfaces *S*_1_ and *S*_2_, respectively. The angle $\theta$ represents the magnitude of the curvature of the surface at a point (Fig. 4).

The neighbourhood normal variance *θ* values for each point were next determined, and then the histogram for the distribution of the *θ* values was generated (Fig. 3b). To reduce human involvement, the optimal segmentation threshold was determined by the Otsu algorithm (the range of segmentation threshold values in this study was 0.8 ~ 1.0 rad). The point clouds for leaves larger than the threshold value were removed, leaving the point clouds of the stem & ear, which were smaller than the threshold value, for the next point cloud clustering.

(3) Ear point cloud clustering

The density of the point clouds is different for wheat canopies of different heights, and the point clouds have clear density characteristics that vary with height (Fig. 3a). Therefore, the density-based spatial clustering of applications with noise (DBSCAN) algorithm, which is based on density features for clustering, was chosen as the algorithm to complete the ear counting. DBSCAN is a density-based spatial clustering algorithm [23]. The density in this algorithm refers to the point density, which is defined as the minimum number of adjacent data points (MinPts) within the specified radius (Eps). The algorithm divides regions with sufficient density into clusters and finds arbitrarily shaped clusters in a spatial database with noise. It defines a cluster as the largest set of densely connected points. Compared to k-means, this algorithm does not require a predeclared number of clusters and is less sensitive to outliers in the data.

**Fig. 5:**
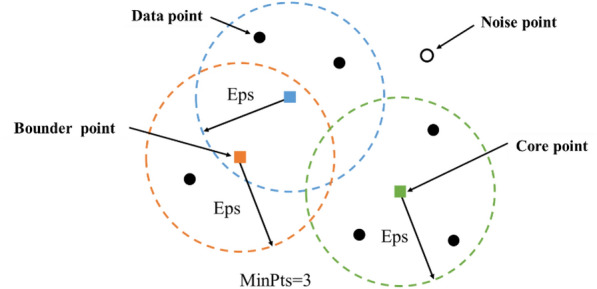
Three types of data points in DBSCAN

The points are divided into three clusters in DBSCAN (Fig. 5) as follows: (1) Core points: the number of points in the Eps neighbourhood of sample point *x*_*i*_ is at least MinPts. (2) Border points: the number of points in the Eps neighbourhood of sample point *x*_*i*_ is less than MinPts; meanwhile, *x*_*i*_ is in the neighbourhood of other core points. (3) Noise point: a point that is neither a core point nor a boundary point.

In the DBSCAN algorithm, the high-density regions that are separated from the low-density regions are grouped as a “cluster”. The flow of the algorithm proceeds as follows:

- An unmarked point is selected randomly as a core point.
- The data points within the specified radius Eps of the core point are grouped. The only parameter that needs to be customized in this algorithm is MinPts, which is the number of points in the neighbourhood of the core point (MinPts were set in the range of 5 to 15 in this study). Eps is the radius of the smallest circular area that encloses all points.
- The above process is repeated until all the data points in the dataset are traversed.

Finally, the number of clusters determined is the number of ears.

#### Voxel-based regional growth

**Fig. 6:**
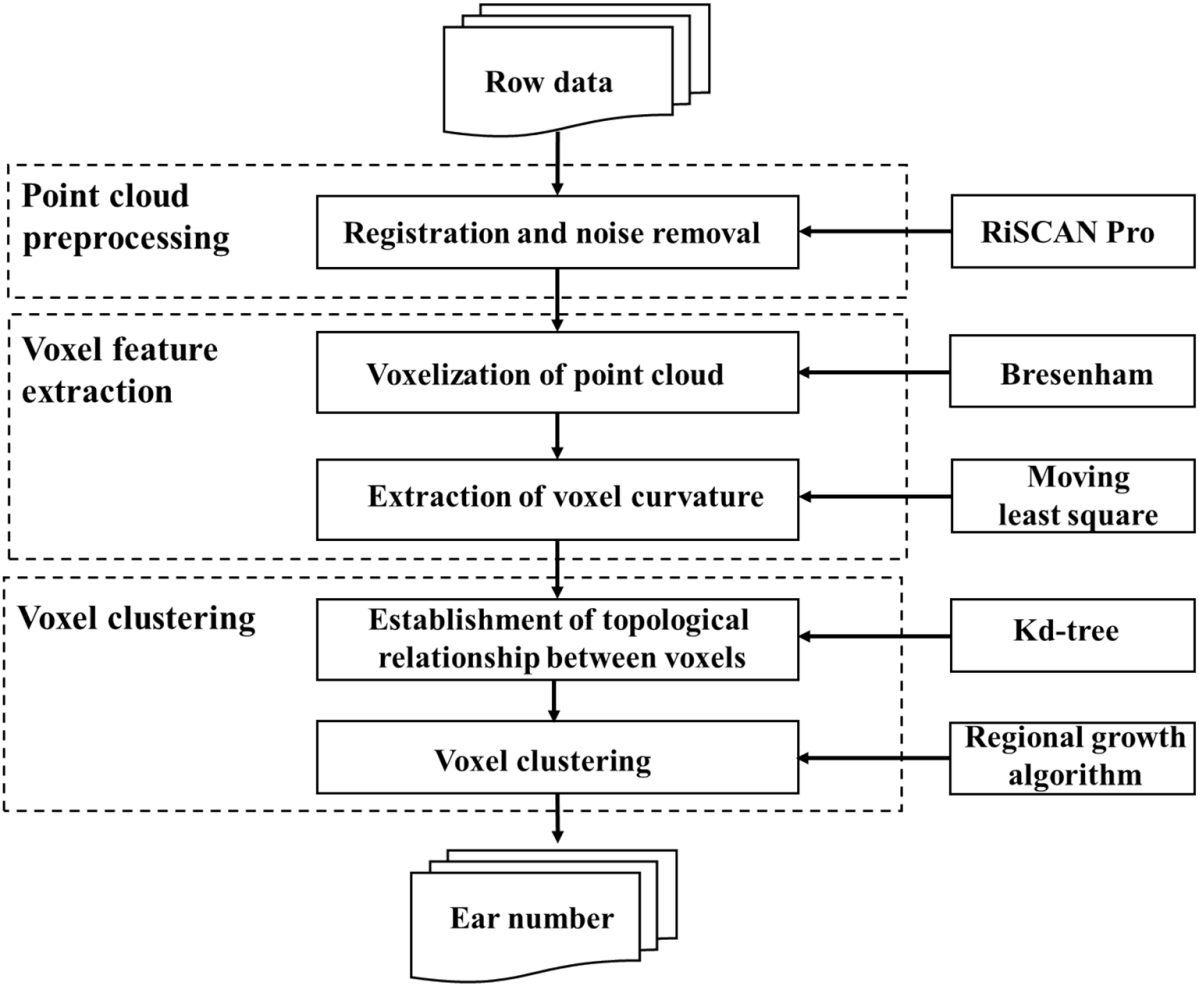
Workflow for estimating the ear number in VBRG

The algorithm for voxel-based regional growth (VBRG) includes three elements, namely, voxelization of the point clouds, establishment of a topological relationship among the voxels, and voxel clustering. First, a voxelization algorithm was used to integrate the point clouds into voxels of regular size and to extract the curvature of the voxel as a feature. Second, a topological relationship among the voxels was established according to the k-dimensional tree (kd-tree), which was composited as a connected area. Finally, a region-growing algorithm was used to divide the voxels into clusters, and the number of clusters was the number of ears (Fig. 6).

1. Point cloud voxelization

**Fig. 7:**
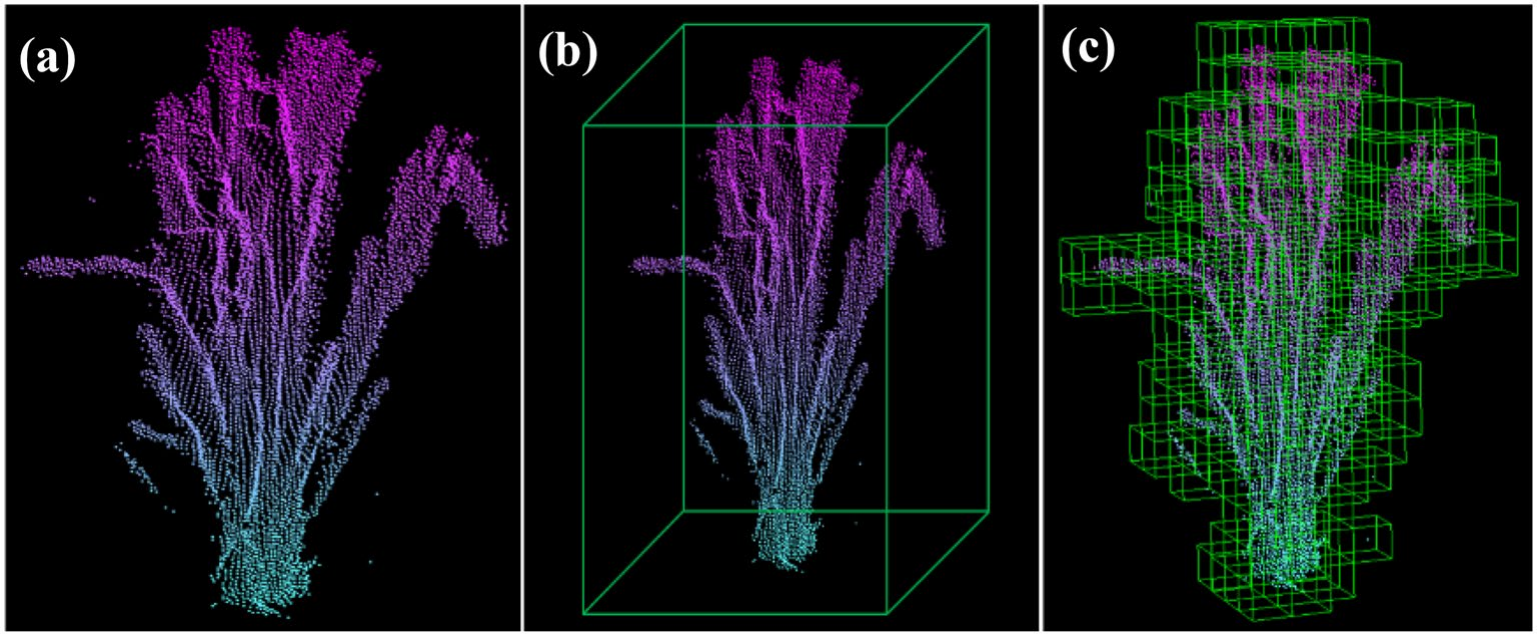
The voxelization process. **a** The point clouds of a single wheat plant, **b** the initial voxel, and **c** the segmented voxels

The concept of the voxel may be considered analogous to the concept of the pixel in the two-dimensional plane. A voxel, the smallest unit of division in three-dimensional space, may be defined as a cube that exists in three-dimensional space. Voxelization is the process of establishing the surface shape of a target object with a finite number of cubes (Fig. 7). Voxelization not only preserves more surface information about the target object but also helps to reduce noise and redundancy in point cloud data pre-processing [24]. The process of point cloud voxelization is as follows:

- Initial voxel creation. First, the maximum coordinate value *P*_max_ (*x*_max_, *y*_max_, *z*_max_) and the minimum coordinate value *P*_min_ (*x*_min_, *y*_min_, *z*_min_) are calculated for all points in the dataset. Second, an initial voxel (space bounding box) is calculated according to *P*_max_ and *P*_min_, where the length, width, and height are given by *x*_max_-*x*_min_, *y*_max_-*y*_min_, and *z*_max_-*z*_min_, respectively.
- Initial voxel segmentation. The initial voxel is divided into small voxels with a specific resolution. The setting of voxel resolution impacts the subsequent algorithm [17]. The use of larger voxels results in a serious loss of detailed information, whereas the use of smaller voxels affects the efficiency of data processing in the algorithm. Currently, there is no clear method for determining the optimal voxel resolution, and it is generally necessary to choose the best value according to the specific object of study [17]. In this study, a voxel resolution of 1 cm in length, width, and height was adopted [13].
- Irrelevant voxel elimination. After completing the voxelization of the point cloud surface, the Bresenham algorithm was used to voxelize the internal space of the point cloud and remove the blank voxels inside the enclosing box of the space. The Bresenham algorithm is the most widely used linear scan conversion method in the field of computer graphics. Expanding to three-dimensional space and taking Pmin as the starting point and each data point as the ending point, we determined the voxel space where the data points were located [25] (Fig. 7).
- Calculation of curvature

(2) Calculation of curvature

In this study, curvature was selected as a characteristic of the voxel according to the morphological characteristics of the wheat stem and leaf. Curvature is an indicator that can be used to measure the degree of unevenness of the geometry [21]. The curvature of a surface can be described with the maximum curvature, the minimum curvature, the mean curvature, the Gaussian curvature, etc. In this study, the mean curvature of the surface constructed by a single voxel interior point was used as the voxel feature. The surfaces were constructed using the moving least squares (MLS) method. MLS does not require meshing of the fitted domain and is suitable for the discrete point model, resulting in smoother and more accurate surfaces [20]. The curvature may be calculated as follows:

- The average curvature *C*_*n*_ of a point* p* is calculated for the surface.
  5 $$2{C}_{n}\overrightarrow{n}=\underset{\mathrm{diam}(A)}{lim} \frac{\nabla A}{A}$$
  where $\overrightarrow{n}$ is the normal vector, $\nabla A$ is an infinitely small region around *p*, and diam (A) is the diameter of this region and is the gradient operator for point *p*.

- The average curvature of *p*_*i*_ was determined by discretizing Eq. (5),
  6 $${C}_{n}\left({p}_{i}\right)=\frac{\overrightarrow{n}}{4{A}_{min}}\gamma$$
  7 $$\gamma =\sum_{j\in {N}_{i}} \left(\mathrm{cot}{\alpha }_{ij}+\mathrm{cot}{\beta }_{ij}\right)\left({p}_{i}-{p}_{j}\right)$$

where *N*_*i*_ is the set that is the diagonal of the $\mathrm{cot}{\alpha }_{ij}$, $\mathrm{cot}{\beta }_{ij }$ which connect the ${p}_{i}$, ${p}_{j}$ edges, respectively.

The determined voxels can be represented as voxel (*x̅*, *y̅*, *z̅*, *C*), where (*x̅*, *y̅*, *z̅*) are the 3D coordinates of the centre of gravity point of the voxels and *C* is the mean curvature of the voxel.

(3) Establishment of the topological relationship among the voxels

Topological relationships should be established for voxels to form connected regions, speed up the inter-neighbourhood search, and improve the algorithm efficiency. A k-dimensional tree (kd-tree) was used to establish the topological relationships between the voxels. The kd-tree is a binary tree that partitions the k-dimensional data space [26]. For 3D space, spatial partitioning between point clouds in three dimensions is needed to create a spatial structure based on the Euclidean distance between the points.

(4) The number of ears as determined by the regional growth algorithm

After the connected regions of the voxels were established, the wheat ear point clouds were extracted based on the regional growth algorithm. The regional growth algorithm is a region-based segmentation algorithm that merges points with similar properties. First, a seed point is designated in an area as the starting point for growth. Second, the points in the field around the seed point are compared with the seed point, and the points with similar properties are merged and continue to grow outwards until no points satisfying the conditions are included. The regional growth algorithm can partition out connected regions with the same characteristics without prior knowledge and can provide boundary information [27–29]. The regional growth of voxels takes the centre of gravity of the voxels as the object and the curvature as the feature of the voxels. The flow of the algorithm proceeds as follows:

- Selection of seed points. The selection of seed points affects the accuracy of segmentation. In this study, the curvature of the voxel was ranked, and the point with the lowest curvature was selected as the initial seed point. What is meant by minimum curvature is that the area where the initial seed point is located is the smoothest. Growth from the smoothest region reduces the total number of regions, improves computational efficiency, and enables the problem of region overlap in the segmentation to be avoided.
- Setting of growth criteria. In this study, the regional growth of the voxels was based on the curvature. The setting of the curvature threshold is crucial, and improper selection can easily cause over-segmentation or under-segmentation. The histogram for the statistical distribution of the voxel curvature was used to calculate the optimal threshold value using the Otsu algorithm. The segmentation thresholds in this study were set to range from 0.3 to 0.8 cm^−1^. The curvature value for each neighbouring point was checked, and the neighbouring points that were less than the curvature threshold were added to the current seed point sequence.
- Condition for setting the termination of growth The growth was terminated when the voxel curvature value was greater than the curvature threshold.

The undefined voxels were then reselected, and the first and second steps were repeated until all voxels were traversed and the number of regions was outputted; this value corresponded to the number of ears.

### Statistical analysis

The agreement between the number of ears computed with the developed methods and the reference ear numbers (manual measurements) was evaluated based on Pearson’s correlation coefficient (*r*) as calculated in Eq. (8). The precision for the number of ears computed with respect to the reference data was also assessed using the root mean square error (RMSE) and relative RMSE (rRMSE), which were calculated using Eqs. (9) and (10) as follows:

8 $$r = \frac{{\mathop \sum \nolimits_{i = 1}^{{N_{plot} }} \left( {y_{i} - \overline{y}} \right)\left( {y_{i}{\prime} - \overline{{y^{\prime}}} } \right)}}{{\sqrt {\mathop \sum \nolimits_{i = 1}^{{N_{plot} }} \left( {y_{i} - \overline{y}} \right)^{2} } \sqrt {\mathop \sum \nolimits_{i = 1}^{{N_{plot} }} \left( {y_{i}{\prime} - \overline{{y^{\prime}}} } \right)^{2} } }}$$

9 $$RMSE = \sqrt {\frac{{\mathop \sum \nolimits_{i = 1}^{{N_{plot} }} \left( {y_{i} - y_{i}{\prime} } \right)^{2} }}{{N_{plot} }}}$$

10 $$rRMSE = \frac{RMSE}{{\overline{y}}} \times 100\%$$

where ${y}_{i}$ and ${{y}_{i}}{\prime}$ are the measured and estimated ear numbers for sample $i$, respectively, $\overline{y }$ and $\overline{{y }{\prime}}$ are the averaged measured and estimated ear numbers over all samples, respectively, and ${N}_{plot}$ is the number of plots. All algorithms in this study were implemented in MATLAB 2016a (MathWorks^®^, USA).

In addition, we used one-way ANOVA to investigate the differences between the estimations of ear numbers with different wheat cultivars, nitrogen fertilization rates, and planting densities in each method. Meanwhile, we also investigated the difference between the estimation results of the two methods. Duncan’s multiple comparisons post hoc test was used to determine differences between stratified means (P < 0.05).

## Results

### Estimation of the number of wheat ears with terrestrial LiDAR

**Fig. 8:**
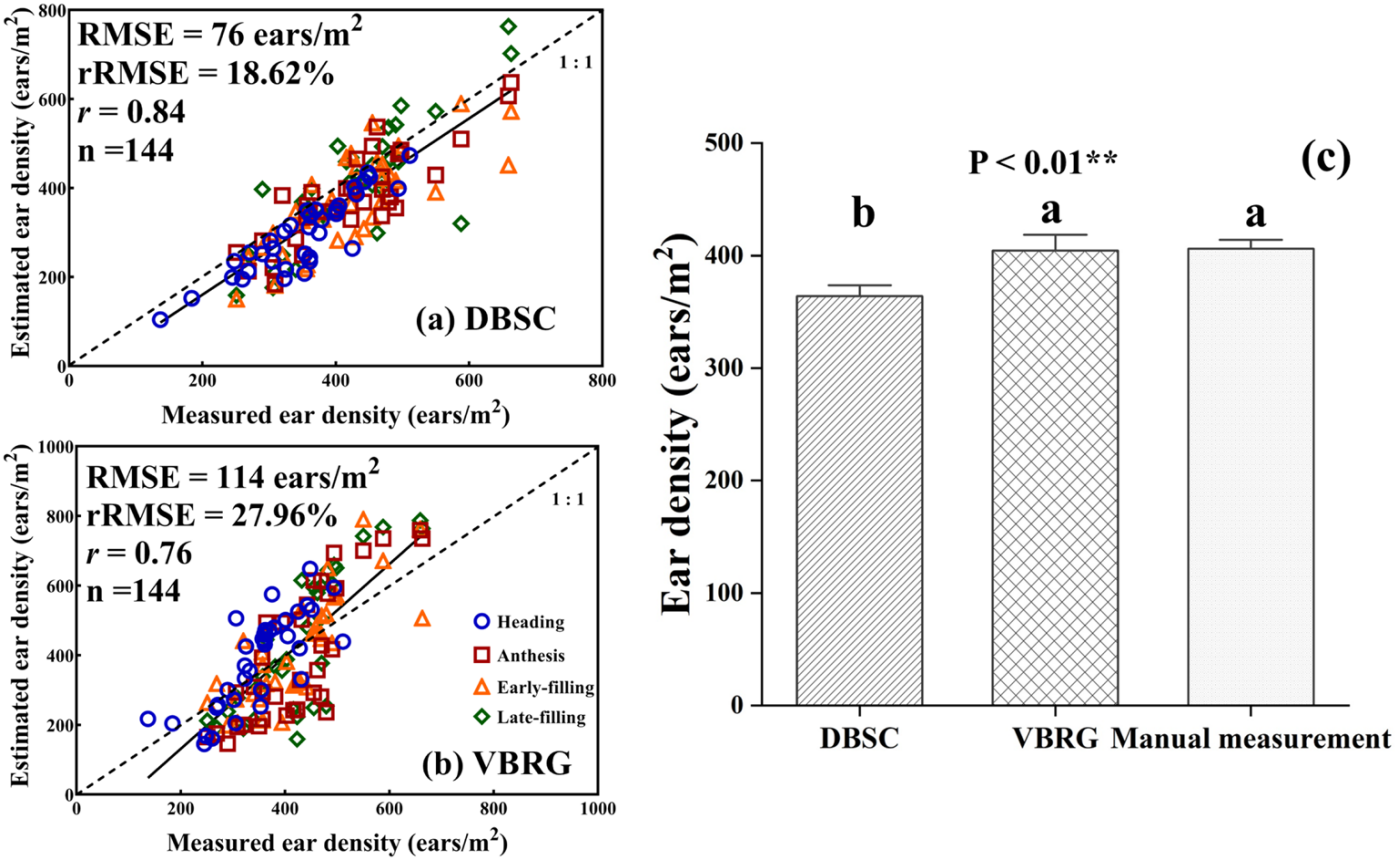
The estimation accuracy of DBSC (**a**) and VBRG (**b**) and analysis of differences among the three estimation methods (DBSC, VBRG, and manual measurement) using an ANOVA test (Significance level: ***P < 0.001, **P < 0.01, *P < 0.05, ns: no significance) (**c**). Different lowercase letters in each group indicate significant differences at P < 0.05

The developed DBSC and VBRG methods can effectively calculate the number of wheat ears in the field (Fig. 8). The DBSC has a high estimation accuracy (RMSE = 76 ears/m^2^, rRMSE = 18.62%, *r* = 0.84) but produces a slight underestimation overall. The VBRG has a low estimation accuracy (RMSE = 114 ears/m^2^, rRMSE = 27.96%, *r* = 0.76), producing an underestimation at low values and an overestimation at high values. In addition, there were significant differences in the results by the three methods (DBSC, VBRG, and manual measurement) (P < 0.01). That is, the results with DBSC were significantly different from those with VBRG and those with manual measurements, while the VBRG and field measurement results were not significantly different.

In addition, the DBSC and VBRG have higher efficiency compared to manual counting (Table 2). The manual counting required 6 h/person to complete ear counts in 36 plots. For the same amount of work, TLS data acquisition and pre-processing takes a total of 2.3 h, and the actual calculation times for DBSC and VBRG are only 0.2 h and 0.4 h, respectively.

**Table 2:** Comparison of time consumption between manual counting, DBSC, and VBRG

Methods	Time (36 plots)	Total time (36 plots)
Manual	6 h/person	6 h/person
DBSC		
TLS scanning	1.6 h	2.5 h
Data pre-processing	0.7 h	
Calculation	0.2 h	
VBRG		
TLS scanning	1.6 h	2.7 h
Data pre-processing	0.7 h	
Calculation	0.4 h	

A total of 4 persons were involved in the manual counting

### Accuracy of the estimated number of ears for the different growth stages

**Fig. 9:**
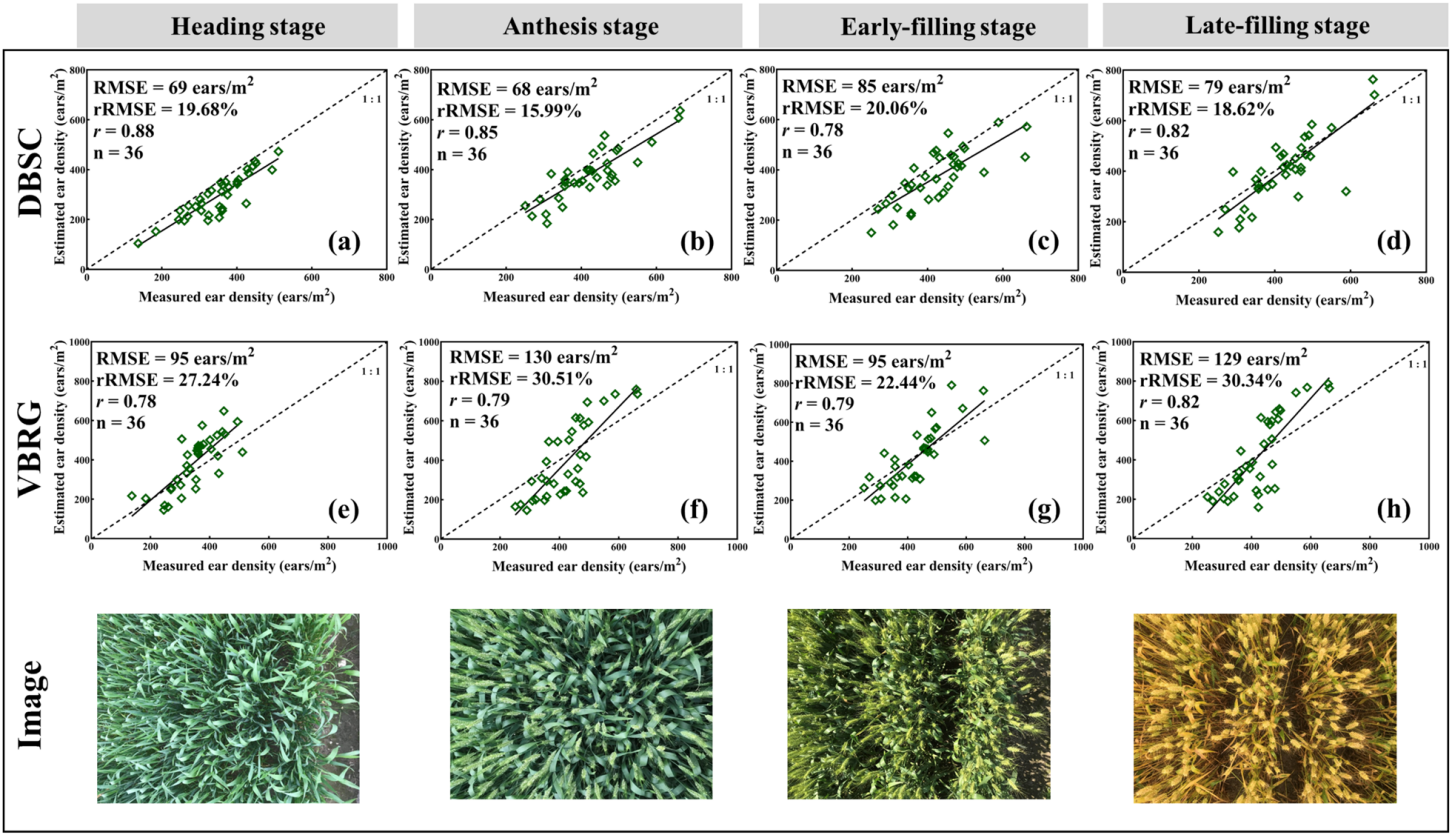
Accuracy of estimation of DBSC and VBRG for various growth stages (heading, anthesis, early-filling, and late filling)

**Fig. 10:**
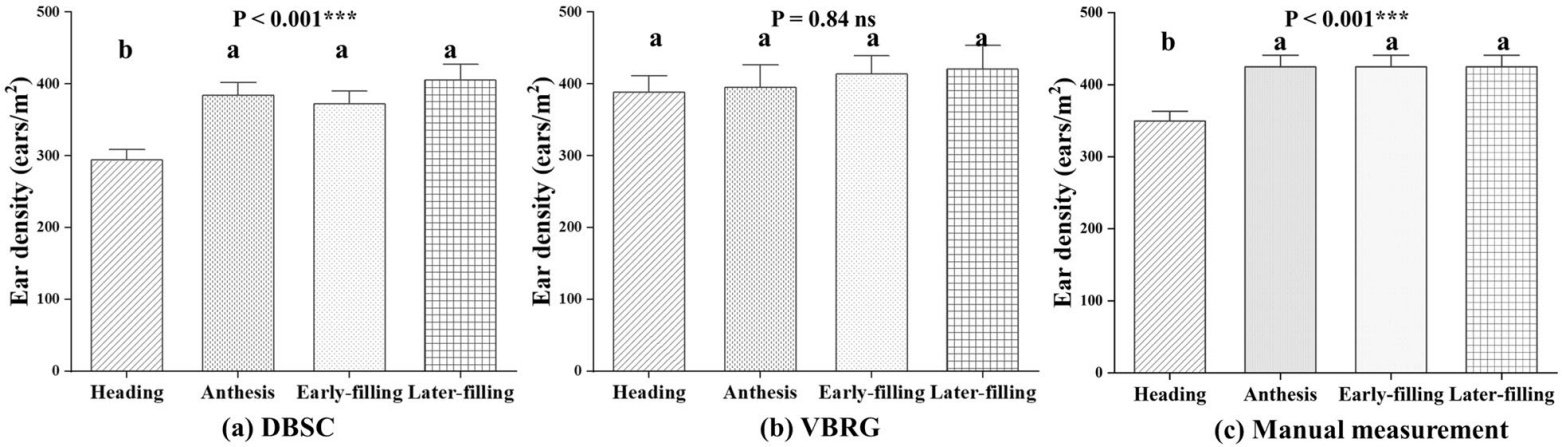
Analysis of the differences between the results of the estimation of different growth stages under different methods (**a** DBSC, **b** VBRG, **c** manual measurement) using an ANOVA test (Significance level: ***P < 0.001, **P < 0.01, *P < 0.05, ns: no significance). Different lowercase letters in each group indicate significant differences at P < 0.05

The accuracies for the estimation of the number of wheat ears by DBSC and VBRG are shown in Fig. 9. For the DBSC method, the accuracy overall gradually increased with the progression of growth stages. The highest accuracy occurred at anthesis (RMSE = 68 ears/m^2^, rRMSE = 15.99%, *r* = 0.85), and the lowest accuracy was found for the early-filling stage (RMSE = 85 ears/m^2^, rRMSE = 20.06%, *r* = 0.78). The underestimation phenomenon gradually decreased with the progression of the wheat growth stages, while overestimation occurred in late filling. For the VBRG method, accuracy was high at the heading and late filling stages and was low at anthesis and the early-filling stage. The highest accuracy occurred at the early-filling stage (RMSE = 95 ears/m^2^, rRMSE = 22.44%, *r* = 0.79), and the lowest accuracy occurred at anthesis (RMSE = 130 ears/m^2^, rRMSE = 30.51%, *r* = 0.79). The estimation accuracy of the DBSC was higher than that of the VBRG for all growth stages. Figure 10 shows the analysis of the differences among the results with the different methods (DBSC, VBRG, and manual measurement) for different growth stages. There were significant differences in the results among different growth stages with both DBSC and manual measurements (P < 0.001). However, there were no significant differences in the results among different growth stages with VBRG.

### Accuracy of the estimated number of ears for the different cultivars

**Fig. 11:**
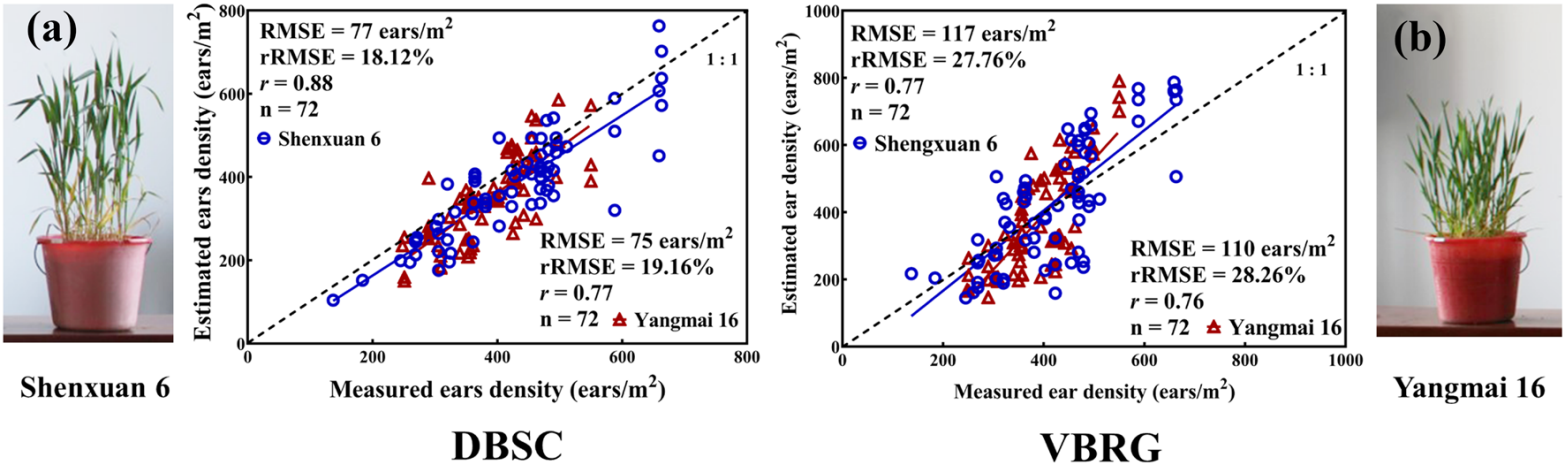
Accuracy of DBSC and VBRG estimation for two cultivars. Indoor images of the plants of the different cultivars (**a** compact type: ‘Shengxuan 6’;** b** diffuse type: ‘Yangmai 16’)

**Fig. 12:**
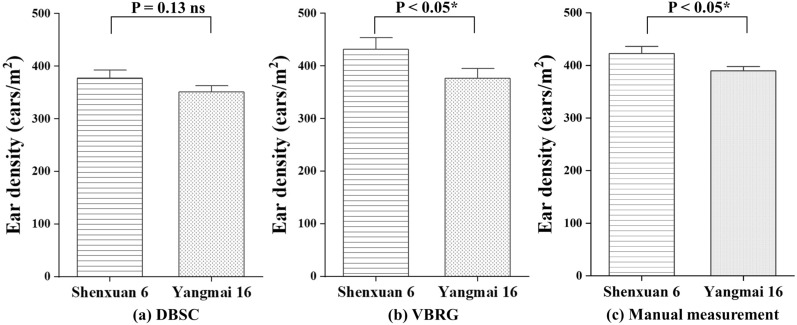
Analysis of the differences between the results of different cultivars (‘Shengxuan 6’ and ‘Yangmai 16’) under different methods (**a** DBSC, **b** VBRG, **c** manual measurement) using an ANOVA test (Significance level: ***P < 0.001, **P < 0.01, *P < 0.05, ns: no significance)

Two cultivars of wheat (‘Shenxuan 6’ is a compact plant and ‘Yangmai 16’ has a more open growth habit) were selected to investigate the possibility of different plant types affecting the accuracies of estimation of the number of ears by the two algorithms. For the DBSC method, the accuracy of the estimation was found to be higher for the compact type (RMSE = 77 ears/m^2^, rRMSE = 18.12%, *r* = 0.88) and lower for the open-habit type (RMSE = 77 ears/m^2^, rRMSE = 18.12%, *r* = 0.88). Similarly, in the case of the VBRG method, the estimation accuracy for the compact type (RMSE = 117 ears/m^2^, rRMSE = 27.67%, *r* = 0.77) was higher than that for the open-habit type (RMSE = 110 ears/m^2^, rRMSE = 28.26%, *r* = 0.76) (Fig. 11). There were significant differences in the results between different cultivars in both the VBRG and manual measurement results (P < 0.05). However, there were no significant differences in the estimates between the two different cultivars with the DBSC method (Fig. 12).

### Accuracy of the estimated number of ears at various N fertilization rates

**Fig. 13:**
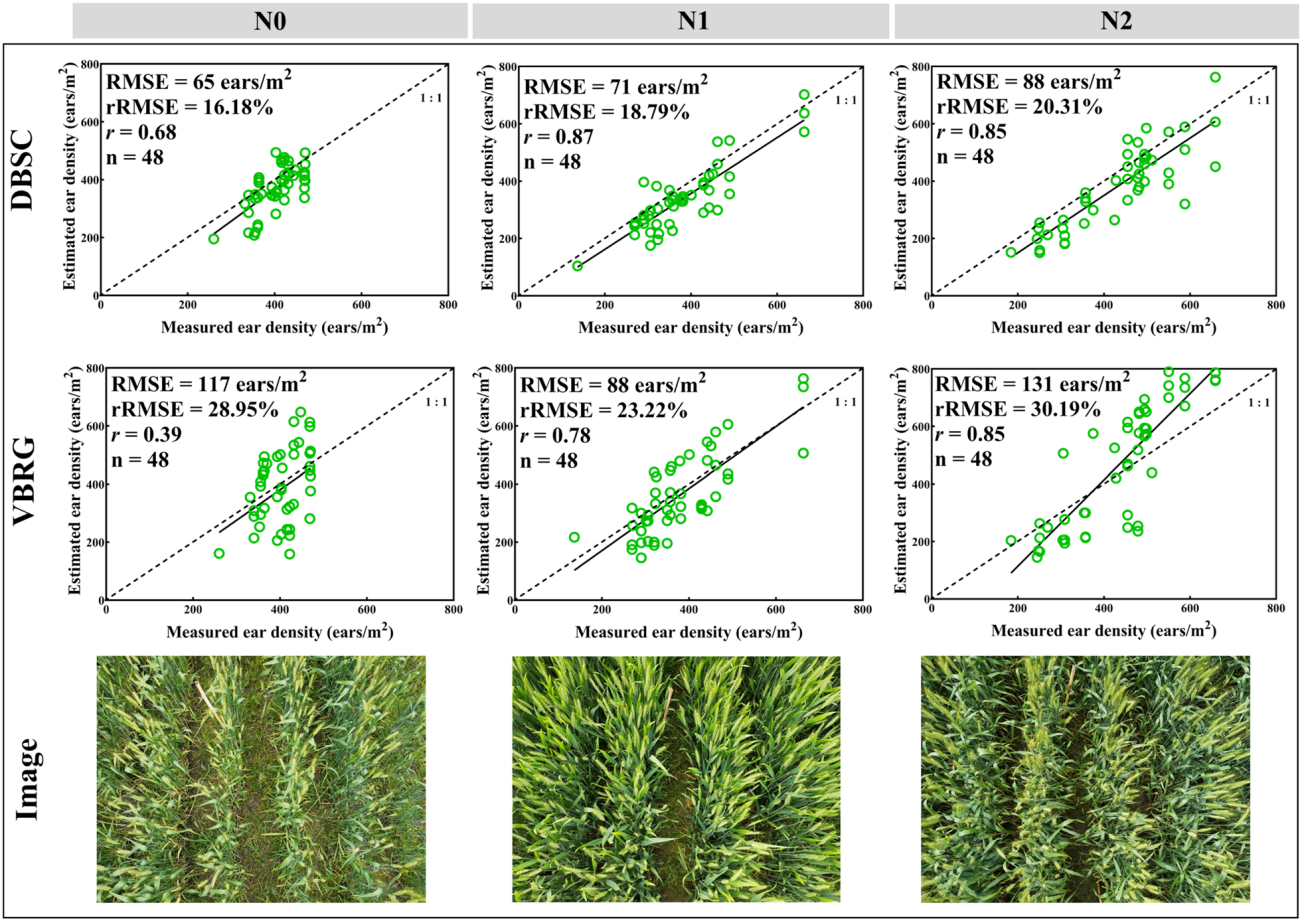
The accuracy of ear number estimation for different N fertilization rates using the DBSC and VBRG methods

**Fig. 14:**
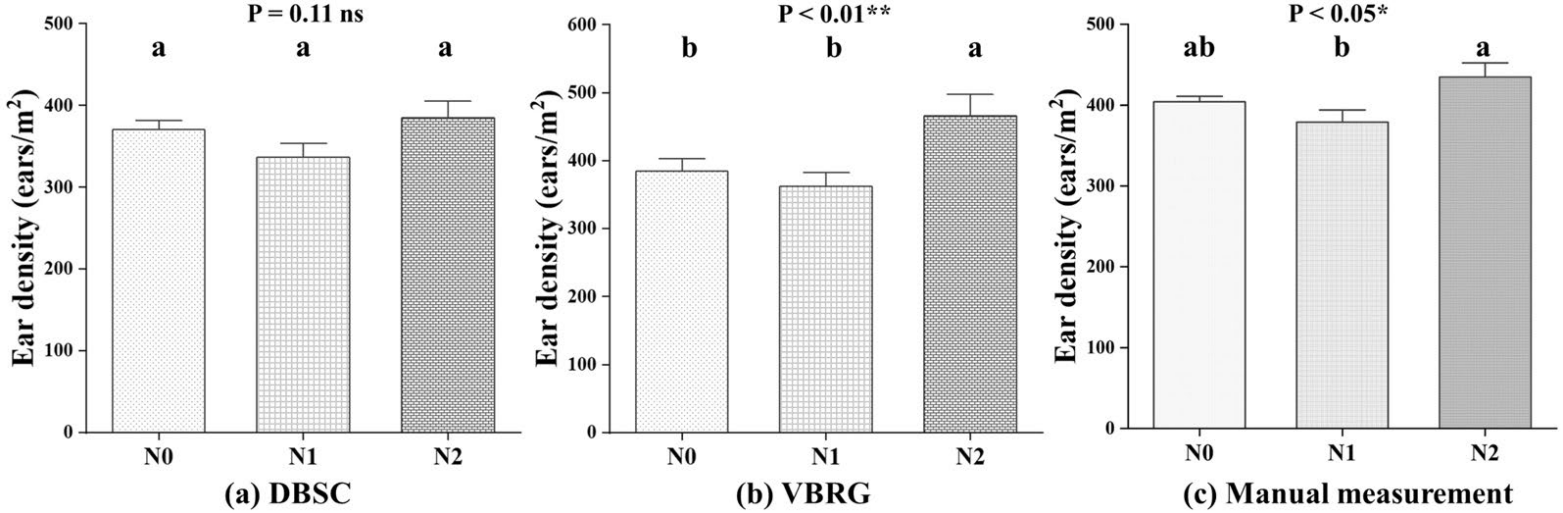
Analysis of the differences between the results of different N fertilization rates under different methods (**a** DBSC, **b** VBRG, **c** manual measurement) using an ANOVA test (Significance level: ***P < 0.001, **P < 0.01, *P < 0.05, ns: no significance). Different lowercase letters in each group indicate significant differences at P < 0.05

Three N fertilization rates (N0, N1, and N2) for the wheat were used to investigate whether the N fertilization rate would affect the accuracies of the estimation of the number of ears based with the DBSC and VBRG methods (Fig. 13). The estimation accuracy decreased with increasing N fertilization rates with the DBSC method, with the highest accuracy being with N0 (RMSE = 65 ears/m^2^, rRMSE = 16.18%). For the DBSC method, the highest accuracy was with N1 (RMSE = 88 ears/m^2^, rRMSE = 23.22%), and the lowest accuracy was with N2 (RMSE = 131 ears/m^2^, rRMSE = 30.19%). There was no significant difference in the estimates among the different N fertilization rates with the DBSC method. However, in the VBRG (P < 0.01) and manual measurements (P < 0.05), there were significant differences in the results among the different N fertilization rates (Fig. 14).

### Accuracy of the estimated number of ears for various planting densities

**Fig. 15:**
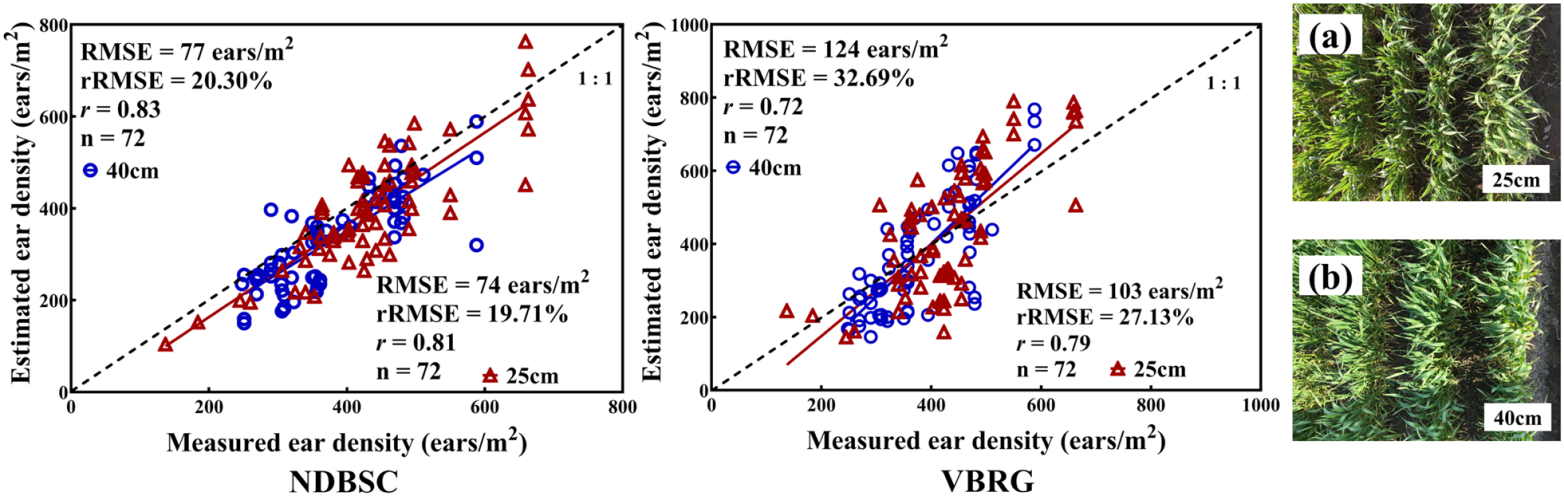
The accuracy of ear number estimation for different planting densities (**a** 25 cm; **b** 40 cm) using the DBSC and VBRG methods

**Fig. 16:**
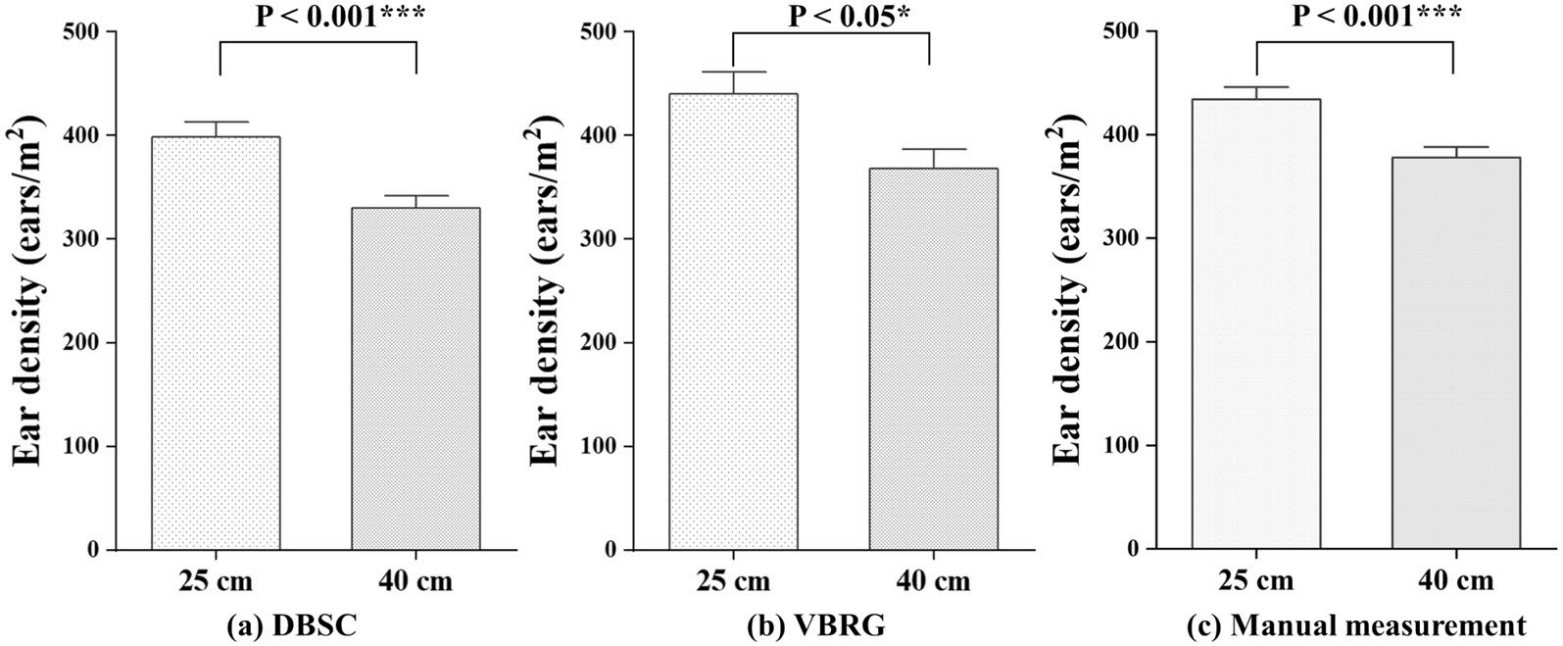
Analysis of the differences between the results for two different planting densities with the three methods (**a** DBSC, **b** VBRG, **c** manual measurement) using an ANOVA test (Significance level: ***P < 0.001, **P < 0.01, *P < 0.05, ns: no significance)

Two planting densities for wheat (20 cm and 40 cm) were used to investigate whether the planting density would affect the accuracies of estimation for the number of ears with the DBSC and VBRG methods. For the DBSC method, the accuracy of the estimation was higher for the 25 cm planting density (RMSE = 74 ears/m^2^, rRMSE = 19.71%) than for the 40 cm planting density (RMSE = 77 ears/m^2^, rRMSE = 20.30%). In the case of the VBRG method, the accuracy of the estimation was also higher for the 25 cm planting density (RMSE = 103 ears/m^2^, rRMSE = 27.13%) than for the 40 cm planting density (RMSE = 124 ears/m^2^, rRMSE = 32.69%) (Fig. 15). In all three methods, DBSC (P < 0.001), VBRG (P < 0.05) and manual measurement (P < 0.001), the results at the two different planting densities were significantly different (Fig. 16).

## Discussion

### Factors affecting the accuracy of DBSC estimation

Compared with the methods of previous studies, the DBSC method makes full use of the structural information of the point clouds based on the morphological differences between the stems and leaves. The segmentation threshold is determined by the Otsu algorithm, which reduces human involvement and improves the generalizability of the algorithm. An algorithm based on differences in density for clustering, known as a density-based clustering algorithm, is a feature of the DBSC method. When the density of the point cloud data is not uniformly distributed, the accuracy of the DBSC method will be affected. The structure of the wheat canopy changes significantly with the growth stage. The spatial heterogeneity of the canopy gradually increases from the heading stage to the late-filling stage, which increases the inhomogeneity of the density distribution of the point cloud data and decreases the accuracy of the estimation of the algorithm (Fig. 9). For density-based clustering, different combinations of the parameters MinPts and Eps have a large impact on the clustering effect [30]. When the spatial density of clusters is not uniformly distributed and the spacing difference is large, the selection of the parameters MinPts and Eps is difficult, resulting in poor clustering quality. The determination of the MinPts values relies on the availability of known reference values, which are empirical and have poor reliability. In future research, new methods for determining the key parameters should be sought to facilitate the automation of the algorithm.

### Factors affecting the accuracy of VBRG estimation

The voxelization in the VBRG method transforms discrete point clouds into connected regions to better exploit the spatial correlation of the point clouds. First, the voxel size has an impact on the accuracy of the algorithm [17]. When the voxel is too large, it contains too many data points, and the loss of detailed information is severe. The choice of voxel size needs to be determined and optimized based on the size and shape of the study object. Second, the selection of voxel features also has an impact on the subsequent algorithmic process. The voxelization process has a smoothing effect. When the point cloud density is high, different types of point clouds are mixed in a single voxel. As a result, different types of point clouds produce only one curvature feature, which results in an underestimation of curvature features. When the point cloud density is low, the curvature features are insignificant, and the resulting eigenvalues are overestimated. Both of these scenarios may reduce the accuracy of the estimation. Furthermore, the threshold values in this study were determined mostly by the Otsu algorithm. If the data distribution does not have significant peaks and valleys, i.e., the data are not well differentiated, the accuracy for the determination of the threshold would be reduced. In other words, voxelization reduces the differentiation between data, which makes the subsequent regional growth algorithm unable to classify accurately, which affects the accuracy of the algorithm. The regional growth algorithm is widely used in 3D point cloud segmentation and clustering because of its simplicity and ease of implementation [31–34]. However, the regional growth algorithm also has some drawbacks, such as being sensitive to noise and being influenced by seed point selection and growth conditions. The sensitivity of the regional growth algorithm to noise arises from the regional growth conditions, i.e., point cloud features such as normal angle and curvature. These curvature features are susceptible to point cloud noise and missing organ point cloud, which may affect the regional growth algorithm. In this study, when the planting density was increased, the amount of point cloud data increased, and the noise also increased, which resulted in a reduction in the accuracy of the estimation (the RMSE increased from 103 ears/m^2^ to 124 ears/m^2^) (Fig. 15). Improving the sensitivity of the growth conditions parameter to noise can greatly enhance the robustness of the algorithm. Principal component analysis can be used to extract the feature vectors of the data, mitigate the effect of noise and improve the operation of the algorithm [34].

### Comparison between DBSC and VBRG

The DBSC method is more suitable for estimating the number of ears of wheat in the field than the VBRG method (Fig. 8). In addition, the DBSC method requires less calculation time and is more suitable for handling large data volumes than the VBRG, which encodes voxels to construct connected components (Table 2) [13]. The DBSC method uses points as the basic unit, which can better represent local point cloud features. Hence, the determined feature vectors are more representative. This method is simple in principle, easy to implement and provides a more accurate characterization of organ growth. However, the computational efficiency of the method decreases with an increase in the number of point clouds and is sensitive to the threshold value that has been set. The VBRG method uses voxels as the basic unit, which reduces data redundancy and computation. However, the size of the voxels is not readily determined and is more sensitive to variations in the internal point density. The method only selects the main features inside the voxels, and some detailed information may be lost. In particular, the iterations of the regional growth algorithm reduce the robustness of the whole algorithm. Overall, the DBSC algorithm (RMSE = 76 ears/m^2^, rRMSE = 18.62%, *r* = 0.84) exhibited a higher accuracy than the VBRG algorithm (RMSE = 114 ears/m^2^, rRMSE = 27.96%, *r* = 0.76) and was more suitable for estimating the number of ears of wheat in the field (Fig. 8).

### Limitations and prospects of DBSC and VBRG

Many studies have measured wheat ear morphology by the colour, texture, edge and orientation characteristics of RGB images [3, 35]. However, the problem of ear shading in three-dimensional space has still not been effectively addressed. Point cloud-based DBSC and VBRG methods have 3D spatial advantages over image recognition. Therefore, such methods could provide an effective solution for the problem of ear shading. Nevertheless, there are some issues with the DBSC and VBRG methods. For example, the problem of missing point clouds in the lower part of the wheat canopy in a field environment leads to a decrease in the estimation accuracy of DBSC and VBRG. Therefore, the TLS scanning site should be continuously optimized during the data acquisition phase to obtain high-quality point cloud data [36]. In addition, more complete wheat point clouds in the field should be generated by fusing point cloud data from TLS and UAV-borne LiDAR [37]. At present, deep learning has been widely used in the classification and recognition of crop organ point clouds. For example, Jin, et al. [38] effectively extracted maize structural phenotypic parameters using TLS data and deep convolutional neural networks. Therefore, optimization of DBSC and VBRG algorithms by deep learning deserves further research.

In addition, we used Otsu twice to distinguish point clouds of uncorrelated, stems, leaves, and ears in the DBSC. Because the ear has a higher height with a denser point cloud when compared with that of leaf and stem in wheat in this study. Otsu has been shown to be effective in distinguishing point clouds of different crop organs [13, 14]. For example, Velumani, et al. [13] distinguished ear and non-ear point clouds of wheat by height based on Otsu. Malambo, et al. [14] also determined the point cloud of green and non-green leaves of sorghum based on Otsu through vegetation indices. However, Otsu has some limitations. For example, the ears are more upright than the leaves for the wheat cultivars in this study, and the ears point cloud can be identified by the different normal vector difference between ears and leaves. However, for some wheat cultivars which have curved ears, the accuracy of ear identification may decrease. Therefore, we need to improve this algorithm so that it could be applied to those cultivars with bending ear. In the future, we can fusion some features of ear volume and reflection intensity of the ear point cloud to reduce the effect of ear bending on the current algorithm.

## Conclusion

In this study, two efficient, non-destructive, and high-throughput algorithms for the automatic calculation of the ear counts for wheat in the field based on terrestrial LiDAR data were proposed and compared. The DBSC method first separates the stem & ear point clouds from the leaf point clouds by the normal difference and then uses a density clustering algorithm to detect the number of ears in the ear point clouds. The VBRG method features voxelized discrete point clouds, constructs intervoxel topologies, and clusters the voxels in connected regions using a regional growth algorithm to calculate the number of ears. The results demonstrated that both algorithms improved accuracy and flexibility for the LiDAR measurement and assessment of the different growth stages, planting densities, and wheat cultivars. The DBSC method performed better than the VBRG method in all aspects. In particular, given that the DBSC method is point-based, it can retain more detailed information and is more suitable for field studies on wheat. Currently, noise and occlusion problems occur when using LiDAR to acquire wheat point clouds because the wheat canopy is low and dense. Since the noise may produce misclassification of ears with the result of overestimation, and the occlusion may lead to underestimation due to miss the ears. It will have a large impact on the methodology of ear recognition here. In the future, we can remove noise through deep learning and reduce occlusion issues through multi-platform LiDAR (e.g. UAV-borne LiDAR and backpack LiDAR). In addition, refinement of the algorithm parameters, especially with respect to selection of the study objects and their morphological characteristics, is a topic that warrants further attention in future research.

## Data Availability

Not applicable.
